# The effects of calcium and glucose supplementation on bone of young female rats in case of disturbances in energy balance caused by their food restriction and exercise

**DOI:** 10.1186/1550-2783-8-S1-P29

**Published:** 2011-11-07

**Authors:** Yuki Aikawa, Yuya Kakutani, Ikuko Ezawa, Naomi Omi

**Affiliations:** 1 Laboratory of Sports Nutrition, University of Tsukuba Graduate School of Comprehensive Human Sciences, Tsukuba 305-8574, Japan; 2 Japan Women’s University, Bunkyo 112-8681, Japan

## Background

Female athletes, with a strong awareness of their weight loss, are prone to restrict their food intake. A major concern arisen from such athletes' daily training would be an imbalance of energy intake and energy expenditure which resulted in an osteoporotic fracture. Calcium (Ca) is a major mineral content in bone, otherwise Glucose (Glu) is an energy source. It is not clear whether Ca or Glu supplementation have a positive effect on bone in case of disturbances in energy balance caused by their food restriction and exercise.

## Methods

49 female Sprague-Dawley rats (age 8 weeks) were divided into 6 groups: ad libitum feeding (0.6% Ca diet) and non-exercise group [Cont group]; ad libitum feeding (0.6% Ca diet) and exercise group [Ex group]; food restriction (0.6% Ca diet)and exercise group [REx group]; food restriction, Ca supplementation (1.2% Ca diet) and exercise group [REx+Ca group]; food restriction (0.6% Ca diet), Glu supplementation and exercise group [REx+Glu group]; food restriction, Ca supplementation (1.2% Ca diet), Glu supplementation, exercise group [REx+Ca+Glu group]. They were reared in individual cages during 38 days. Food restriction was 70% of food intake of the Cont group. Exercise was voluntary wheel running. We measured the number of revolutions every day. After the treatment period, intra-abdominal fat, femur, lumbar spine and tibia were collected. Statistical analysis was performed using ANOVA followed by a Scheffe’s post hoc comparisons test (p<0.05).

## Results

Final body weight of REx group (167.4±10.2g), REx+Ca group (172.5±18.9g) and REx+Ca+Glu (229.6±15.4g) group compared with the Cont group (257.5±12.5g) were significantly lower (p<0.001). Running distance was not significant different among the 5 groups (EX group , REx group, REx+Ca group, REx+Glu group and REx+Ca+Glu group) (7083±5575, 12021±7392, 10750±7266, 10743±6182 and 9144±6048 m). Abdominal fat weight of EX group (2.05±0.86g/100gBW), REx group (1.26±0.49g/100gBW), REx+Ca group (1.12±0.63g/100gBW), REx+Glu group (1.72±0.46g/100gBW) and REx+Ca+Glu group (1.56±1.05g/100gBW) compared with the Cont group (4.67±1.56g/100gBW) were significantly lower (p<0.001). Femur weight and femur length of REx group (0.431±0.029g and 3.151±0.067cm) and REx+Ca (0.454±0.045g and 3.175±0.082cm) group compared with the Cont group (0.543±0.030g and 3.417±0.039cm) were significantly lower (p<0.001).

## Conclusions

It is concluded that Ca supplementation had no effect, but Glu supplementation had a positive effect on bone under food restriction and wheel running.

